# Esophageal Perforation in Zollinger–Ellison Syndrome: A Scoping Review of Management and Outcomes

**DOI:** 10.1002/jgh3.70323

**Published:** 2026-01-22

**Authors:** Agostino Fernicola, Domenico Parmeggiani, Felice Crocetto, Armando Calogero, Alessio Cece, Domenico Romano, Giacomo Benassai, Gennaro Quarto, Michele Santangelo

**Affiliations:** ^1^ Department of Advanced Biomedical Sciences, Unit of Emergency Surgery Federico II University Naples Italy; ^2^ Department of Integrated Activities in Surgery, Orthopedy and Hepato‐Gastroenterology University of Campania “Luigi Vanvitelli” Naples Italy; ^3^ Department of Neurosciences, Sciences of Reproduction and Odontostomatology University Federico II Naples Italy; ^4^ ASL Naples 1, San Paolo Hospital Center Naples Italy; ^5^ Department of Clinical Medicine and Surgery Federico II University Naples Italy

**Keywords:** acid hypersecretion, Boerhaave syndrome, esophageal perforation, gastrinoma, Zollinger–Ellison syndrome

## Abstract

Esophageal perforation in Zollinger–Ellison syndrome (ZES) is an exceedingly rare and life‐threatening manifestation of gastrinoma‐related acid hypersecretion. While peptic ulceration and severe reflux are recognized complications of ZES, perforation represents the ultimate consequence of uncontrolled hyperacidity. Evidence remains confined to isolated case reports with no prior systematic synthesis. A scoping review was conducted in accordance with PRISMA‐ScR and the Joanna Briggs Institute framework. A comprehensive search of PubMed, Embase, Scopus, and Web of Science was performed from database inception to September 2025. Eligible studies included any report describing esophageal perforation in confirmed or suspected ZES. Iatrogenic perforations were excluded. Data were extracted on patient demographics, clinical presentation, diagnostic modalities, management strategies, and outcomes. **S**even eligible cases published between 2001 and 2025 were identified. Patients were aged 44–63 years (4 males, 3 females). The distal esophagus was affected in approximately 70% of cases, usually after chronic peptic injury; three patients (≈43%) presented with spontaneous Boerhaave‐type rupture. Computed tomography was diagnostic in all cases, and endoscopy was used in six. Surgical repair with mediastinal drainage or T‐tube repair was the mainstay of management, while conservative therapy failed. Endoscopic stenting achieved successful leak control in one patient, whereas overall survival across all cases was approximately 86%, with one death due to delayed diagnosis and sepsis. Esophageal perforation in ZES represents a predictable but preventable endpoint of chronic acid injury. Rapid imaging, decisive surgical or endoscopic repair, and definitive acid suppression are essential to survival. Awareness of this rare complication among surgeons and gastroenterologists may facilitate early recognition and improve outcomes.

## 1 Introduction

Zollinger–Ellison syndrome (ZES) is a rare but clinically significant disorder characterized by gastrin‐secreting neuroendocrine tumors (gastrinomas) that cause pathological gastric acid hypersecretion [1]. The resulting acid load leads to refractory peptic ulcer disease, severe gastroesophageal reflux, and mucosal injury throughout the upper gastrointestinal tract [1, 2]. Although duodenal and jejunal ulcerations represent the hallmark lesions of ZES, the esophagus is not immune to the effects of chronic acid exposure [2, 3]. Severe reflux esophagitis, peptic strictures, and Barrett's metaplasia have been documented in up to 10%–15% of patients [4]. Yet, esophageal perforation remains an exceptional and life‐threatening manifestation of the disease.

Esophageal perforation in the setting of ZES represents the extreme endpoint of unchecked hypersecretory injury [1, 3]. Its occurrence underscores the destructive potential of acid hypersecretion when mucosal protection and timely diagnosis fail [1, 3]. While spontaneous or iatrogenic esophageal rupture is well recognized in general surgical practice, the contribution of gastrinoma‐induced acid hypersecretion to perforation is poorly understood [1, 2]. The literature on this topic is limited to isolated case reports, each emphasizing different aspects of presentation, diagnosis, and management. As a result, the true clinical spectrum, underlying mechanisms, and prognostic determinants remain obscure.

The rarity of this condition poses diagnostic and therapeutic challenges. Clinical presentation may mimic more common entities such as Boerhaave syndrome, perforated ulcer, or mediastinitis of other origin [1, 2, 3, 4, 5, 6, 7, 8]. Moreover, delayed recognition is frequent, as the underlying hypergastrinemic state is often unrecognized at the time of rupture [1, 2, 5, 6]. Once established, perforation carries a high risk of sepsis and mortality, making early suspicion and appropriate management crucial [1, 2, 6]. Understanding the cumulative experience from published cases is therefore essential to delineate characteristic patterns and guide evidence‐informed care [9].

Given the absence of prior systematic or scoping reviews on this topic, we conducted a comprehensive scoping review to identify and map all published cases of esophageal perforation occurring in patients with ZES. Our aim was to describe the clinical presentations, proposed pathogenic mechanisms, diagnostic approaches, management strategies, and outcomes reported in the literature. By synthesizing this highly fragmented evidence, we sought to clarify the pathways leading to perforation in ZES and to highlight diagnostic and therapeutic considerations for surgical and gastroenterological practice. To our knowledge, this is the first scoping review systematically synthesizing all published cases of esophageal perforation in the setting of ZES.

## 2 Methods

### 2.1 Protocol and Registration

This scoping review was conducted in accordance with the Preferred Reporting Items for Systematic Reviews and Meta‐Analyses extension for Scoping Reviews (PRISMA‐ScR) guidelines and the methodological framework proposed by Arksey and O'Malley (2005) as refined by the Joanna Briggs Institute [10].

The protocol was prospectively registered and is publicly accessible through the Open Science Framework (OSF) under the identifier *DOI: 10.17605/OSF.IO/CA3H2*.

### 2.2 Objectives and Review Question

The objective of this review was to systematically map and describe all published cases of esophageal perforation occurring in patients with ZES, focusing on clinical presentation, diagnostic approach, management strategy, and outcome.

The guiding review question was: “What are the clinical characteristics, underlying mechanisms, diagnostic modalities, management strategies, and outcomes of esophageal perforation in patients with Zollinger–Ellison syndrome?”

### 2.3 Eligibility Criteria

All published reports in English describing esophageal perforation in confirmed or suspected ZES were eligible, including single case reports, case series, or descriptive cohort studies. No restrictions were applied regarding patient age, sex, language, geographic location, or publication date.

Exclusion criteria were: (1) perforations of iatrogenic origin (e.g., endoscopic, surgical, or traumatic); (2) reports without sufficient clinical detail to confirm ZES or to characterize the perforation; (3) narrative reviews or conference abstracts lacking primary data.

When ambiguity arose, inclusion was determined by consensus among the reviewers.

### 2.4 Information Sources

A comprehensive electronic search was performed across PubMed/MEDLINE, Embase, Scopus, and Web of Science databases, from January 2000 to September 2025. The search was supplemented by manual screening of the reference lists of all included articles and relevant reviews to identify additional eligible studies. Gray literature, including theses and non‐indexed journals, was not systematically searched due to the highly specific clinical context and rarity of the condition.

### 2.5 Search Strategy

Search terms combined controlled vocabulary (MeSH and Emtree) with free‐text keywords related to ZES, gastrinoma, and esophageal perforation. An example of the PubMed search string was: (“Zollinger‐Ellison Syndrome”[Mesh] OR “Zollinger‐Ellison” OR “gastrinoma”) AND (“Esophageal Perforation”[Mesh] OR “esophageal rupture” OR “Boerhaave” OR “esophageal fistula” OR “esophagus perforation”).

The strategy was adapted for each database to ensure comprehensive coverage. All retrieved citations were imported into Rayyan for de‐duplication and screening.

### 2.6 Selection of Sources of Evidence

Two independent reviewers (AF and a secondary investigator [AC]) screened titles and abstracts for relevance. Full‐text articles were then assessed against inclusion criteria. Disagreements were resolved by discussion and, when necessary, consultation with a senior reviewer (MS).

The entire selection process was documented in a PRISMA‐ScR flow diagram summarizing numbers of records identified, screened, excluded, and included.

### 2.7 Data Charting Process

Data extraction (charting) was performed independently by two reviewers using a pre‐piloted form developed in Microsoft Excel. Extracted variables included:

Bibliographic information (author, year, country); patient demographics (age, sex); clinical presentation and mechanism of perforation; site and extent of the perforation; diagnostic modalities employed; management strategy (surgical, endoscopic, conservative); postoperative course and outcome; follow‐up duration.

Where information was incomplete, details were recorded as “not reported.” No assumptions or imputations were made.

### 2.8 Data Synthesis

Data were synthesized descriptively in line with the objectives of scoping review methodology. No quantitative meta‐analysis was planned due to the heterogeneity and small number of cases.

Findings were summarized narratively and organized thematically according to presentation, diagnostic strategy, treatment approach, and clinical outcome.

Aggregated frequencies and proportions were calculated when data allowed, and results were tabulated to illustrate key patterns.

### 2.9 Ethical Considerations

Because this review was based entirely on previously published data, no ethical approval or patient consent was required. The study adhered to the principles of transparency, reproducibility, and open science as outlined in the OSF registration.

## 3 Results

### 3.1 Study Selection

**FIGURE 1:**
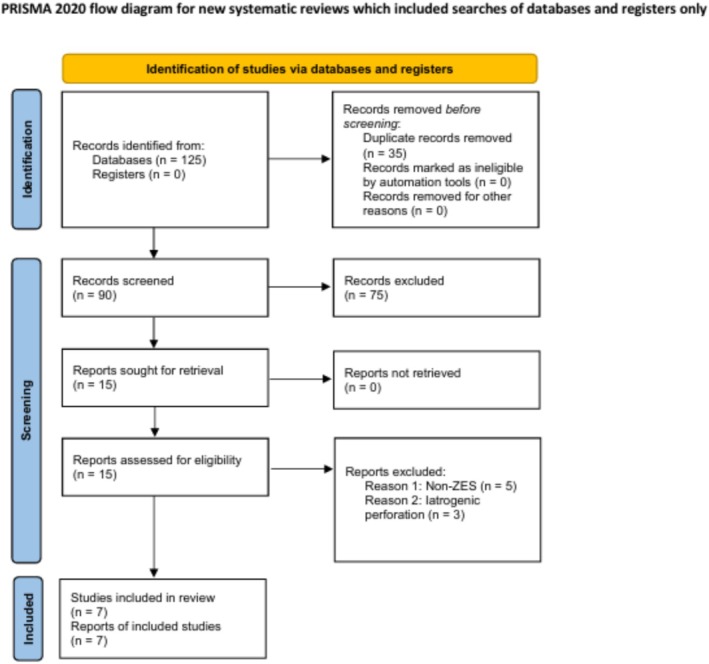
PRISMA‐ScR flow diagram illustrating the process of study identification, screening, eligibility assessment, and final inclusion of reports describing esophageal perforation in ZES. A total of 125 records were identified through database searches, 35 duplicates were removed, 90 titles and abstracts were screened, 15 full‐text articles were assessed for eligibility, and 8 studies met the inclusion criteria.

A total of 125 records were identified through electronic database searches (PubMed, Embase, Scopus, and Web of Science). After removal of 35 duplicates, 90 titles and abstracts were screened. Seventy‐five studies were excluded for irrelevance to the topic or absence of esophageal involvement. Fifteen full‐text articles were assessed for eligibility, of which eight were excluded: five because no ZES diagnosis was confirmed, and three because the perforation was iatrogenic in nature. Ultimately, seven studies describing seven individual cases met the inclusion criteria and were included in the review. The study selection process is summarized in the PRISMA‐ScR flow diagram (Figure 1).

### 3.2 Clinical Presentation and Diagnosis

The seven included cases spanned over two decades (2001–2025) and involved patients aged 44–63 years, with a male‐to‐female ratio of approximately 5:3. In three cases, the perforation represented the initial presentation of an unrecognized gastrinoma, highlighting that esophageal rupture can occasionally be the sentinel event revealing ZES. The clinical presentation was typically acute. Patients reported severe retrosternal or epigastric pain, vomiting, or dysphagia; in several cases, fever and hemodynamic instability suggested sepsis or mediastinitis. Radiological imaging, most frequently contrast‐enhanced computed tomography, was diagnostic in all cases, showing pneumomediastinum, extraluminal contrast leak, or fluid collections. Endoscopy was performed in six of the seven cases (≈85%) to confirm mucosal ulceration and to exclude other causes such as malignancy or foreign body perforation. Laboratory evaluation, when reported, revealed markedly elevated fasting gastrin levels consistent with ZES.

### 3.3 Management Strategies

Surgical intervention was the predominant approach, reflecting the severity and life‐threatening nature of the condition. Primary repair with or without mediastinal drainage was performed in four to five patients, depending on report completeness. Two patients underwent T‐tube repair followed by delayed definitive surgery for gastrinoma removal or acid control. In one patient, gastrectomy was ultimately required due to recurrent peptic damage and intractable acid secretion. An endoscopic approach was reported in a single case, involving temporary covered stent placement for leak control. The patient survived, although a persistent esophago‐mediastinal fistula required prolonged management. Only a single case was treated conservatively, but this strategy was unsuccessful, underscoring the limited role of non‐operative therapy in this clinical context.

### 3.4 Outcomes and Patterns of Disease

Overall outcomes were favorable. Six of seven patients survived, corresponding to an approximately 86% survival rate. Mortality occurred in one patient with delayed diagnosis and septic shock. The distal esophagus was the most frequent site of perforation (five cases, ~70%), followed by mid‐esophageal (two cases) and proximal (one case) locations. Perforation mechanisms included chronic peptic injury (five cases, ~70%) and spontaneous Boerhaave‐type rupture (three cases, ~43%). No clear relationship emerged between the anatomical site of perforation and patient outcome, but earlier recognition and surgical management were consistently associated with recovery. The median follow‐up duration, when reported, ranged from 6 months to 1 year, with no recurrences or late complications described apart from a single stricture requiring dilation. Comprehensive case characteristics and summarized findings are presented in Table 1 and Table 2. To allow a consistent comparison across published reports, we systematically summarized the clinical manifestations, acid‐suppression status, biochemical findings, and evidence of gastrinoma for each patient (Table 3). This aggregation highlights the heterogeneity of diagnostic confirmation in the existing literature, with several cases meeting only partial criteria for ZES prior to perforation.

**TABLE 1:** Characteristics of published cases of esophageal perforation in patients with ZES, 2001–2025.

Author (Year)	Age/Sex	Clinical presentation	Site of perforation	Diagnostic method	Management	Clinical outcome
Ng (2001) [11]	56/M	Epigastric pain, sepsis	Distal (peptic)	Contrast radiography + endoscopy	Surgical closure + mediastinal drainage	Survived, good recovery
Shahshahan (2017) [12]	47/M	Post‐prandial vomiting, chest pain	Boerhaave‐type (mid‐lower)	Chest CT	Primary surgical repair + drainage	Survived
Ruttan (2012) [5]	49/M	Vomiting, thoracic pain	Boerhaave‐type (mid‐esophagus)	CT	Endoscopic stent placement	Survived after prolonged fistula management
Shaik (2023) [9]	44/M	Chest pain, fever	Distal (peptic)	CT + endoscopy	T‐tube repair → PPI + gastrinoma resection	Survived, asymptomatic at 1 year
Ito (2023) [4]	55/F	Dysphagia, chest pain	Distal/necrotizing peptic	Endoscopy	Resection → subsequent dilation	Survived, benign stricture
Robinson (2024) [6]	58/F, 63/M	Chest pain, hematemesis	Distal/aorto‐gastric fistula	CT + endoscopy	Emergency surgery/drainage	1 death, 1 survival
Vittori (2025) [13]	51/F	Epigastric pain, fever	Distal (peptic)	CT + endoscopy	T‐tube repair → gastrectomy	Survived, good outcome at 6 months

Abbreviations: CT = computed tomography; PPI = proton‐pump inhibitor.

**TABLE 2:** Summary of aggregated findings from published cases of esophageal perforation in ZES.

Category	Key findings	Frequency (*n*)	Percentage (%)
Mechanism of perforation	Spontaneous (Boerhaave‐type)	3	~43
	Peptic (chronic acid injury)	4	~57
Anatomical site	Distal esophagus	5	~70
	Mid esophagus	1	~15
	Proximal esophagus	1	~15
Diagnostic modality	CT scan	7	100
	Endoscopy	6	~85
Management	Surgical repair (closure, drainage, T‐tube)	5	~71
	Endoscopic (stent placement)	1	~14
	Conservative therapy	1	~14
Outcome	Survived	6	~86
	Died	1	~14

Abbreviation: CT = computed tomography.

**TABLE 3:** Evidence supporting Zollinger–Ellison syndrome diagnosis in reported cases with esophageal perforation.

Author (Year, case)	Signs/symptoms before perforation	Anti‐secretory therapy before perforation	Diagnosis of ZES before perforation	Erosive esophagitis	↑ Esophageal acid exposure	↑ Gastric acid secretion	↑ Serum gastrin	Evidence of neuroendocrine tumor
**Ng (2001)** [11]	4 years: diarrhea, duodenitis, severe esophagitis, dysphagia, long distal stricture	**Yes** (PPI)	**No** (ZES diagnosed post‐op)	**Yes**	NR	NR	**Yes** (> 2000 pg/mL)	**Yes** (pancreatic gastrinoma)
**Shahshahan (2017)** [12]	> 1‐year abdominal pain, vomiting; severe esophagitis; multiple duodenal ulcers	**NR** (PPI implied but not stated)	**No**	**Yes** (severe ulcerative esophagitis)	NR	NR	**Yes** (784 pg/mL off‐PPI)	**Yes** (duodenal bulb gastrinoma, Octreoscan+)
**Ruttan (2012)** [5]	3 days vomiting + hematemesis, hypotension; history of probable ZES, gastric ulcers, esophageal strictures with dilatations	**No** (only oxycodone reported)	**Yes (**probable ZES in past records)	**Yes** (peptic strictures related to reflux/ZES)	NR	NR	NR	**No** (no NET documented)
**Shaik (2023)** [9]	Long‐standing GERD; prior perforated gastric ulcer; chest discomfort, nausea, repeated bilious vomiting, unable to swallow secretions	**Yes** (pantoprazole 40 mg BID)	**No** (ZES diagnosed during admission)	**NR** (perforation described, stomach normal; esophagitis not detailed)	NR	NR	**Yes** (433 pg/mL)	**Yes** (metastatic well‐differentiated NET on liver biopsy)
**Ito (2023)** [4]	5‐year refractory GERD; prior esophageal rupture; necrotizing esophagitis; severe lower esophageal stricture	**Yes** (long‐term PPI for GERD)	**No** (gastrinoma found at esophagectomy)	**Yes** (necrotizing esophagitis, severe peptic damage)	NR	NR	**Yes** (1200 → 510 pg/mL post‐op)	**Yes** (duodenal G1 NET, gastrin+)
**Robinson (2023) – Case 1** [6]	Acute nausea, vomiting, abdominal pain; hx suspected ZES, severe esophagitis, gastritis, duodenitis, recurrent GI bleeds	**NR**	**Yes** (suspected ZES, lost to follow‐up)	**Yes** (severe esophagitis in history)	NR	NR	NR	**Yes** (duodenal bulb gastrinoma with liver mets at autopsy)
**Robinson (2023) – Case 2** [6]	Vomiting → sudden pleuritic chest pain; hx MEN1, prior distal pancreatectomy for gastrinoma, chronic esophageal stricture with dilatations	**NR**	**Yes** (MEN1 with prior gastrinoma)	**NR** (stricture described, esophagitis not specified)	NR	NR	NR	**Yes** (previous pancreatogenic gastrinoma)
**Vittori (2025)** [13]	Nausea, vomiting, intractable abdominal pain, melena; prior perforated duodenal ulcer with known prepyloric gastrinoma	**Yes** (pantoprazole 80 mg BID, high‐dose IV PPI)	**Yes** (known prepyloric gastrinoma/ZES)	**Yes** (Los Angeles grade D esophagitis)	NR	NR	**Yes** (> 2000 pg/mL)	**Yes** (1.5 cm G2 gastrinoma, 4/18 LN+)

Abbreviations: BID = bis in die; GERD = gastroesophageal reflux syndrome; NR = not reported; PPI = proton pump inhibitors; ZES = Zollinger–Ellison Syndrome.

## 4 Discussion

This scoping review systematically synthesizes seven published cases of esophageal perforation associated with ZES, spanning from 2001 to 2025.

Despite their rarity, these cases provide valuable insight into the interplay between chronic hypergastrinemia, mucosal vulnerability, and mechanical stress that culminates in full‐thickness esophageal rupture. The uniformity of certain clinical and radiologic features across independent reports strongly suggests a reproducible pathogenic sequence, rather than a coincidental association.

### 4.1 Pathophysiological Interpretation

ZES results from gastrin‐secreting tumors, most commonly pancreatic or duodenal gastrinomas, that cause continuous acid hypersecretion [1, 2, 3]. Prolonged exposure of esophageal mucosa to this hyperacidic environment leads to chronic esophagitis, ulceration, and ultimately to transmural weakening [1, 2, 3, 14]. In classical ZES, peptic complications dominate the duodenal and jejunal regions, but esophageal injury has been reported in up to 10%–15% of cases, ranging from severe reflux esophagitis to stricture formation [1, 2, 3, 14]. Perforation, however, represents the far end of this continuum, an event so rare that fewer than 10 cases have been documented over more than two decades. It is also noteworthy that severe erosive esophagitis is far more common in advanced gastroesophageal reflux disease (GERD) than in ZES; yet, spontaneous perforation in GERD is exceedingly rare and typically occurs only in the context of forceful emesis or instrumentation [15]. This contrast suggests that in ZES, the additive effect of extreme, sustained acid hypersecretion on chronically injured mucosa may create a distinct susceptibility to full‐thickness rupture, although the limited number of cases prevents any definitive comparison.

The reviewed cases illustrate two overlapping mechanisms of rupture. The first, and perhaps most common, is chronic peptic ulceration of the distal esophagus, seen in the reports of Ng et al., Ito et al., and Vittori et al., in which persistent acid exposure produces progressive ulcerative necrosis and mural fragility [4, 11, 13]. Over time, even minor barometric or mechanical stress may precipitate transmural perforation. The second mechanism is acute barotrauma, exemplified by Shahshahan et al., and Ruttan et al., where vigorous vomiting against a closed glottis leads to abrupt pressure spikes that tear the already compromised esophageal wall, reproducing the Boerhaave‐type rupture [5, 12]. The anatomical clustering of perforations in the distal third of the esophagus (five of seven cases, approximately 70%) is consistent with this dual mechanism: the distal segment experiences the highest acid load and intraluminal pressure gradient. Notably, in several patients the perforation constituted the initial presentation of previously unrecognized ZES, emphasizing how this syndrome may remain clinically silent until catastrophic decompensation occurs.

### 4.2 Diagnostic Considerations

Diagnosis in all reports depended on rapid cross‐sectional imaging, with CT being the single most decisive tool. CT scans identified extraluminal air, mediastinal fluid, and leakage of oral contrast in every case, confirming its indispensability. In the cases reported by Shaik et al. and Vittori et al., CT also revealed subtle wall thickening and perigastric fluid consistent with chronic acid injury, supporting the hypothesis of peptic origin [9, 13]. Endoscopy was used in six of the seven cases (≈85%), offering direct confirmation of mucosal ulceration or visible perforation. In some instances, endoscopy was performed postoperatively or after stabilization to avoid exacerbating mediastinal contamination. Endoscopic findings of deep ulceration with surrounding inflammation, as described by Ito et al., were virtually pathognomonic when paired with hypergastrinemia [4].

Laboratory evaluation provided crucial corroboration. Fasting gastrin levels were elevated to several times the upper reference limit in every case where measurement was available. Some patients also underwent secretin stimulation testing, confirming the presence of autonomous gastrin secretion. Importantly, biochemical confirmation was frequently achieved only after stabilization, underscoring the principle that source control takes precedence over etiologic workup in acute perforation. Nonetheless, the combination of high serum gastrin and peptic mucosal damage should immediately prompt consideration of ZES in any unexplained esophageal rupture.

### 4.3 Therapeutic Strategies

Management strategies reflected both the general tenets of esophageal perforation care and the special demands imposed by ZES physiology. Prompt surgical repair remained the cornerstone of therapy, applied in five to six of the seven patients [16]. Primary closure with mediastinal or pleural drainage achieved definitive healing when performed within 24 h of symptom onset, as seen in Ng et al. and Shahshahan et al. [11, 12]. In delayed cases or where the tissue was severely inflamed, T‐tube repair served as a controlled fistulization technique, allowing decompression and gradual healing [9, 13]. This approach is particularly useful in friable, acid‐damaged tissue where primary closure might not hold. One patient underwent total gastrectomy for refractory hypersecretion and recurrent ulceration, illustrating the need for definitive acid control in selected cases [13].

Endoscopic therapy was described in a single report, in which a temporary covered stent was placed to control the leak [5]. Although the patient survived, the course was complicated by a persistent fistula that required prolonged management. While such endoscopic interventions may be feasible in carefully selected cases with contained perforation, their use in ZES‐related esophageal rupture remains anecdotal, and surgical repair should remain the standard of care. Purely conservative management, attempted in one patient, was unsuccessful, underscoring that non‐operative treatment has virtually no role in this context, particularly when hyperacidic gastric effluent perpetuates contamination [4].

Across all cases, rigorous acid suppression was a universal therapeutic pillar. High‐dose proton pump inhibitors were initiated in every patient, often continued indefinitely. Several reports noted the adjunctive use of somatostatin analogs (octreotide) to curb gastrin secretion, and in select patients, surgical resection of the gastrinoma was performed after recovery from perforation. This dual approach (mechanical repair followed by hormonal control) is crucial, as persistent hypergastrinemia risks recurrent ulceration or re‐perforation.

### 4.4 Outcomes and Prognostic Patterns

The collective survival rate was approximately 86% (six of seven patients). The sole death occurred in the report by Robinson et al., where delayed diagnosis and widespread mediastinal sepsis proved fatal despite surgical intervention [6]. Time to diagnosis consistently emerged as the single most decisive prognostic factor. No clear correlation was observed between perforation site and outcome, but early source control and comprehensive acid management were common denominators of survival. Follow‐up data, though inconsistently reported, indicated durable healing and return to oral alimentation in all survivors. One patient developed a benign stricture requiring endoscopic dilation, while others remained asymptomatic at 6–12 months of follow‐up [5].

An interesting secondary observation concerns the interaction between acid hypersecretion and surgical healing. Several authors, notably Shaik et al. and Vittori et al., observed accelerated mucosal healing once acid suppression was optimized postoperatively, supporting the hypothesis that continuous acid exposure delays granulation and epithelial regeneration [9, 13]. Conversely, persistent acid reflux, if inadequately managed, may undermine even technically sound repairs. This emphasizes the unique postoperative vulnerability of ZES patients compared to those with idiopathic Boerhaave's syndrome.

### 4.5 Implications and Perspectives

Clinically, these findings should prompt heightened vigilance among surgeons and gastroenterologists managing ZES. Awareness is particularly important because patients with non‐ZES erosive esophagitis rarely progress to perforation, implying that atypical or severe ulcerative disease should always prompt consideration of a hypersecretory state. Any sudden onset of chest or upper abdominal pain, dysphagia, or sepsis in such patients warrants immediate CT evaluation to exclude perforation. When diagnosed early, timely operative repair combined with aggressive acid suppression can transform an otherwise catastrophic event into a survivable complication. Long‐term care must include endocrine follow‐up, biochemical surveillance of gastrin levels, and assessment for recurrent or metastatic gastrinoma. The high postoperative survival observed across cases supports the view that ZES‐associated perforation is not inherently fatal, provided that diagnosis and acid control are prompt. Given the increasing recognition of ZES‐related esophageal injury, multidisciplinary care involving gastroenterologists, surgeons, and endocrinologists is pivotal for both acute management and long‐term disease control.

### 4.6 Preventive Strategies and Future Perspectives

Beyond acute management, prevention and long‐term control are equally important to mitigate recurrence and chronic complications. Although esophageal perforation in ZES remains exceedingly rare, its occurrence highlights the importance of early recognition and acid control [17]. Preventive measures should begin with prompt identification of ZES in patients presenting with refractory or multiple peptic ulcers, severe or atypical gastroesophageal reflux, or esophagitis unresponsive to standard proton pump inhibitor (PPI) therapy [17]. Screening for fasting hypergastrinemia and gastric acid output in such patients could allow diagnosis before irreversible mucosal damage occurs [18]. In established ZES, rigorous acid suppression, adherence to therapy, and regular endoscopic surveillance of the esophagus may prevent progression to ulceration and perforation [17]. Surgeons and gastroenterologists should maintain a high index of suspicion for hypergastrinemic states when faced with recurrent upper gastrointestinal perforations or refractory esophagitis [17, 18].

Recent pharmacologic developments offer the potential for improved long‐term acid control and potential reduction in perforation risk [19]. Next‐generation PPIs and potassium‐competitive acid blockers (P‐CABs), such as vonoprazan, may offer more potent and sustained acid suppression compared with conventional PPIs, and could be advantageous in patients with ZES who are refractory to standard therapy [20, 21]. In parallel, long‐acting somatostatin analogs (e.g., lanreotide, octreotide, pasireotide) reduce gastrin secretion and hormone‐driven tumor activity, thereby lowering acid output [20, 22]. Although direct evidence in ZES is limited to small series and case reports, these agents remain promising adjuncts in the chronic management of gastrinoma‐associated hypersecretion. Their incorporation into postoperative or maintenance regimens may further help prevent recurrent ulceration or esophageal injury. Based on the collective evidence, Table 4 summarizes possible pragmatic clinical approaches. Future studies should evaluate standardized protocols integrating endocrine, surgical, and pharmacologic management, and assess outcomes under modern antisecretory therapy. Multicenter collaboration and registry‐based data collection could clarify long‐term recurrence rates and optimal timing for tumor resection.

**TABLE 4:** Suggested clinical management strategies for different stages of ZES.

Clinical stage	Recommended actions
Suspected ZES (refractory peptic disease, severe GERD)	Measure fasting gastrin, perform secretin test, start high‐dose PPI or P‐CAB
Confirmed ZES without complications	Long‐term acid suppression ± somatostatin analog; regular endoscopic surveillance
Esophageal ulceration or early perforation	Urgent imaging (CT), early surgical or endoscopic repair, lifelong acid suppression
Post‐repair follow‐up	Endocrine assessment for gastrinoma resection, biochemical monitoring, proton pump blockade maintenance

## 5 Limitations

The limitations of this review mirror those of its primary data. All included studies are case reports, susceptible to publication bias, incomplete reporting, and heterogeneity in diagnostic detail. There is an absence of standardized data regarding perforation size, timing, contamination severity, and exact acid‐suppressive regimens. Nevertheless, the reproducibility of findings across independent sources and the biologic plausibility of the underlying mechanisms support the coherence of the observed pattern. Future progress will require multicenter registries capturing rare upper gastrointestinal emergencies in the setting of neuroendocrine tumors, allowing for meaningful aggregation and analysis. Moreover, functional follow‐up data are almost entirely lacking in current literature, such as swallowing recovery and quality‐of‐life outcomes.

## 6 Conclusion

Esophageal perforation in ZES, though exceedingly rare, was documented in seven published cases over two decades. The typical patient was a middle‐aged adult with unrecognized or poorly controlled gastrinoma. CT imaging was diagnostic in all cases, and early operative or endoscopic repair combined with definitive acid suppression achieved survival in approximately 86% of patients. Awareness among surgeons and gastroenterologists remains essential for timely diagnosis and optimal outcomes.

## Funding

This work was supported by Università degli Studi di Napoli Federico II.

## Conflicts of Interest

The authors declare no conflicts of interest.

## Data Availability

Data sharing not applicable to this article as no datasets were generated or analysed during the current study.
